# CEA-delta could be a biomarker of tumor phenotype, clinical stage, and chemotherapeutic response in rectal cancer with OCT4-positive cancer stem cells

**DOI:** 10.3389/fonc.2023.1258863

**Published:** 2023-09-07

**Authors:** Ivan David Lozada-Martinez, Maria Paz Bolaño-Romero, Lina Lambis-Anaya, Yamil Liscano, Amileth Suarez-Causado

**Affiliations:** ^1^ Grupo Prometheus y Biomedicina Aplicada a las Ciencias Clínicas, Department of Biochemistry, School of Medicine, Universidad de Cartagena, Cartagena, Colombia; ^2^ Grupo de Investigación en Salud Integral (GISI), Departamento Facultad de Salud, Universidad Santiago de Cali, Cali, Colombia

**Keywords:** rectal neoplasms, human POU5F1 protein, carcinoembryonic antigen, prognosis, neoplasm staging, drug therapy

## Abstract

**Background:**

There is very limited evidence on biomarkers for evaluating the clinical behavior and therapeutic response in rectal cancer (RC) with positive expression of cancer stem cells (CSCs).

**Methods:**

An exploratory prospective study was conducted, which included fresh samples of tumor tissue from 109 patients diagnosed with primary RC. Sociodemographic, pathological and clinical characteristics were collected from medical records and survey. The OCT4 protein was isolated using the Western Blot technique. It was calculated the ΔCEA, ΔOCT4, and ΔOCT4/GUSB values by assessing the changes before and after chemotherapy, aiming to evaluate the therapeutic response.

**Results:**

Patients had an average age of 69.9 years, with 55% (n=60) being male. Approximately 63.3% of the tumors were undifferentiated, and the most frequent staging classification was pathological stage III (n=64; 58.7%). Initial positive expression was observed in 77.1% of the patients (n=84), and the median ΔCEA was -1.03 (-3.82 - 0.84) ng/ml, with elevated levels (< -0.94 ng/ml) found in 51.4% of the subjects (n=56). Being OCT4 positive and having an elevated ΔCEA value were significantly associated with undifferentiated tumor phenotype (p=0.002), advanced tumor progression stage (p <0.001), and negative values of ΔOCT4 (p <0.001) (suggestive of poor therapeutic response) compared to those without this status.

**Conclusion:**

This study identified a significant and directly proportional association among the values of ΔCEA, ΔOCT4, and ΔOCT4/GUSB. These findings suggest that ΔCEA holds potential as a clinical biomarker for determining the undifferentiated tumor phenotype, advanced clinical stage, and poor therapeutic response in RC with CSCs positive expression.

## 1 Introduction

Colorectal cancer (CRC) is one of the cancers associated with the highest burden of disease worldwide, with approximately 2 million new cases diagnosed and nearly 1 million deaths reported annually (1–3). It is considered the third most common malignancy and the second most deadly on a global scale. According to expert projections, it is estimated that by the year 2040, both the incidence and mortality rates of CRC will double, posing a significant threat to healthcare systems worldwide (2). The behavior of this disease varies, being more aggressive in regions where the Western diet is prevalent and disparities in healthcare access exist (4). Consequently, various international organizations emphasize the importance of addressing CRC as a priority in global public health and emphasize the need for research to aid in the timely identification and treatment of this malignancy (5–7). This research should utilize innovative resources like translational research (8) and precision medicine (9).

Rectal cancer (RC) specifically represents a more aggressive subtype of CRC, characterized by rapid progression and an unfavorable phenotype compared to other colon locations (10–12). Genetic intratumour heterogeneity (gITH) underlies this phenomenon, resulting from the interplay between genetic and epigenetic variations (tumor biology) and transcriptional plasticity (13). This culminates in population-specific phenotypic plasticity (14). Consequently, extrapolating the effects of chemotherapy treatments for RC management across populations with distinct genetic and epigenetic characteristics poses significant challenges, explaining the variations in response rates and treatment success (13, 14). Therefore, understanding the tumor biology of each population becomes imperative to implement precision medicine and identify valuable biomarkers. Cancer stem cells (CSCs) act as a foundation for tumor growth, metastasis, and therapy resistance (15). They represent an intriguing target in translational cancer research, enabling personalized treatment for public health priority cancers. Nevertheless, the variation in gene expression of CSCs in RC remains poorly studied.

Octamer-binding transcription factor 4 (OCT4) is a critical protein involved in the self-renewal of stem cells and is expressed during embryonic phases. In adults, OCT4 has been associated with numerous malignancies as a marker of CSCs expression (16). Tumors expressing OCT4 may exhibit a more aggressive behavior, with rapid evolution and adaptability to the tumor microenvironment, resulting in a poor chemotherapeutic response. Some previous studies have mainly explored OCT4’s clinical significance in terms of clinical staging, progression, and chemotherapeutic response (17–19). However, these studies primarily focus on CRC rather than RC. Moreover, biomarkers capable of identifying OCT4 expression in tumors, which could be valuable in clinical practice, have not yet been investigated.

The oncofetal antigen known as carcinoembryonic antigen (CEA) is expressed in colorectal epithelial cells and serves the purpose of intercellular adhesion and cell recognition (20). Its origin lies in the embryonic endodermal epithelium of the fetus, and its expression is regulated by oncofetal genes (oncofetal antigens are proteins which are typically present only during fetal development but are found in adults with certain kinds of cancer) (21–27), among which OCT4 could be linked. Following birth, traces of CEA persist in colorectal tissue, yet its levels in the bloodstream are minimal. CEA has been identified as a prognostic biomarker for CRC due to its increased expression and secretion into the blood during invasion and tissue damage (28). Nevertheless, the relationship between CEA and oncofetal genes in terms of affinity and sensitivity remains unclear. Establishing the association between CEA and OCT4 would provide valuable evidence for the identification of gITH and phenotypic plasticity, facilitating personalized approaches and chemotherapy treatments for RC based on clinical progression and chemotherapeutic response. Therefore, considering the global significance of identifying dependable and reproducible biomarkers for RC diagnosis and prognosis, as well as supporting the development of precision medicine treatments, this study aimed to explore the association between serum CEA behavior, tumor phenotype, clinical evolution and chemotherapeutic response in individuals with RC while considering OCT4 status.

## 2 Methods

### 2.1 Patient selection and sample collection

An exploratory prospective study was conducted, which included fresh samples of tumor tissue and from 109 patients diagnosed with primary RC who were diagnosed at two tertiary referral centers in the city of Cartagena, Colombia, from 2018 to 2023.

Patients included in the study met the following criteria: 1) They agreed to participate by providing informed consent and donating the sample during the diagnostic colonoscopy or follow-up (before any chemotherapy treatment for the first sample); 2) Their complete medical history was available to verify the data obtained during the interview; 3) They attended evaluation, follow-up, and treatment by oncology and oncological surgery departments; 4) They had undergone at least 2 sessions of chemotherapy; 5) The medical records contained documented CEA values before and after the chemotherapy sessions; 6) They have not undergone curative or palliative surgery. Patients with a different diagnosis of primary gastrointestinal cancer, who have previously received chemotherapy and those who reported any of the following conditions around the time of blood sample collection for CEA analysis were excluded: 1) Surgical or non-surgical acute gastrointestinal disease (diverticulitis, inflammatory bowel disease, ulcer, pancreatitis); 2) Liver diseases; and 3) Chronic obstructive pulmonary disease. All samples met the appropriate size criteria for molecular analysis. Prior to any treatment, the first sample was obtained during the diagnostic colonoscopy, and the second sample was acquired during the follow-up colonoscopy subsequent to the chemotherapy sessions. The collected fresh tissues were embedded in RNAlater™ and stored at -80°C for subsequent analysis.

### 2.2 Data collection and variables

Sociodemographic, pathological and clinical characteristics were collected from medical records and structured survey. In this study, the tumor tissues were evaluated by expert pathologists using hematoxylin and eosin slides and documented in the pathological analysis reports.

Data such as age, sex, main symptom and clinical history, tumor differentiation grade, lymph node involvement, presence of metastasis, and TNM stage were collected. In the study, tumors with characteristics described as moderately or poorly differentiated were considered undifferentiated. The description of clinical-pathological staging followed the subcategories of stages (Tis [0], I, IIA, IIB, IIC, IIIA, IIIB, IIIC, IVA, IVB, IVC) based on the eighth edition of the American Joint Committee on Cancer (AJCC) staging system (29). Pathological staging, however, encompassed only the four major groups (I, II, III, IV). Clinical staging was determined by the stage system and the histopathological report, categorizing tumors as local/early, regional/advanced, or metastatic/advanced. Hence, the progression status was defined as early or advanced (regional or metastatic). CEA levels were obtained from the medical records (The CEA value after chemotherapy was derived from the average of all CEA values within 6 months after the completion of at least 2 chemotherapy sessions), and the delta was calculated as follows: CEA before chemotherapy - CEA after chemotherapy = ΔCEA. In accordance with previous studies that have established cut-off scores (30, 31), a reference value of 0.94 was utilized (elevated ΔCEA for < -0.94 and normal ΔCEA for > -0.94). In this case, the elevated ΔCEA value suggests progression and poor therapeutic response following chemotherapy. In addition, ΔOCT4 and ΔOCT4/GUSB were calculated to evaluate the response in cancer stem cell marker expression after chemotherapy. GUSB was used as a control for the validity of the molecular techniques (western blot). These calculations followed the same equation as ΔCEA (pre-chemotherapy value - post-chemotherapy value). Thus, a negative ΔOCT4 and ΔOCT4/GUSB values indicate increased expression following chemotherapy, which will be interpreted as a poor therapeutic response.

### 2.3 Western blot analysis

The OCT4 protein was isolated using the Western Blot technique. The tissues were thawed and resuspended in lysis buffer [20 mM Tris-HCl (pH 7.4), 150 mM NaCl, 10% glycerol, 0.2% Nonidet P-40, 1 mM EDTA, 1 mM EGTA, 1 mM phenylmethylsulfonyl fluoride (Sigma-Aldrich, St. Louis, MO, USA), 10 mM NaF, 5 mg/ml aprotinin (Sig-ma-Aldrich), 20 mM leupeptin (Sigma-Aldrich, St. Louis, MO, USA), and 1 mM sodium orthovanadate (Sigma-Aldrich, St. Louis, MO, USA)]. The concentration of total protein in the supernatant was quantified using a spectrophotometric method (32). The absorbance at 595 nm was calculated using a standard curve previously prepared with bovine serum albumin (BSA). The samples were prepared with Laemmli loading buffer and denatured by heating at 95°C for 5 minutes. 30 μg of protein were loaded onto a 10% polyacrylamide gel prepared with sodium dodecyl sulfate (SDS-PAGE) and subjected to electrophoresis in the presence of an electrophoresis buffer at a constant voltage (33). After electrophoresis, the proteins were transferred from the gel to a PVDF membrane (iBlot™ Transfer Stack, PVDF Invitrogen™ Thermo Waltham, MA, USA) using the iBlot™ 2 Gel Transfer Device dry transfer technology (Thermo Scientific™, Waltham, MA, USA). Ponceau staining was performed to confirm that the transfer was successful. Subsequently, the membrane was treated with a blocking solution of 5% skim milk in TTBS1X [10 mM Tris/HCl, 150 mM NaCl, 0.05% Tween-20 (pH 7.5)], and then incubated overnight with the primary antibody anti-OCT4 diluted 1:2000 (Abcam, Cambridge, UK) [EPR2054] (ab109183). After the incubation period, the membrane was washed with TTBS 1X to remove excess antibody. The membrane was then incubated with a horse-radish peroxidase-conjugated secondary antibody for 2 hours. Finally, the membrane was washed with TTBS 1X and the immunodetection was performed using the Super-Signal™ West Pico chemiluminescent substrate (Thermo Scientific™, Waltham, MA, USA). The results were validated using anti-beta glucuronidase antibody 1:2000 (Abcam Cambridge, UK) [EPR10616] (ab166904) as a housekeeping antibody. The PVDF mem-branes were analyzed using an imaging documentation system, using the iBright CL1000 equipment (Thermo Scientific™ Waltham, MA, USA). The iBright analysis software desktop version (Thermo Scientific™ Waltham, MA, USA) was used to measure the band densitometry to determine OCT4 expression.

### 2.4 Statistical analysis

The normality of quantitative variables was tested using the Kolmogorov–Smirnov test. Data were presented as mean ± standard deviation (SD) for continuous variables and median (interquartile, IQR) for skewed variables. Qualitative variables were summarized using frequency and percentages. Comparative analysis was carried out using Pearson’s Chi-square test or Fisher’s exact test for categorical variables and Mann–Whitney or Kruskal-Wallis test for quantitative variables. To identify the variables associated with ΔCEA, an unadjusted logistic regression was conducted. Furthermore, an exploratory analysis was carried out using linear regression to ascertain any potential association between the quantitative values of ΔOCT4 and ΔOCT4/GUSB with ΔCEA. In addition, the calculation of the prevalence ratio (PR) was performed to evaluate the strength of the association between categorical variables and the outcome of ΔCEA across different subgroups. Pearson’s or spearman’s correlation coefficient tests were used for the evaluation of potential correlations between the ΔOCT4 and ΔOCT4/GUSB level of expressions and ΔCEA. A p-value <0.05 was considered statistically significant. All analyses were performed using Statistical Package for the Social Sciences (SPSS) version 29.0 software.

### 2.5 Ethical statements

This study was approved by the Ethics Committee of the Universidad de Cartagena (Minutes No. 108, 10 May 2018), and was conducted in accordance with the principles of the Helsinki Declaration. Each eligible participant signed an informed consent form.

## 3 Results

### 3.1 Patients’ characteristics

The patients had an average age of 69.9 years, with 55% (n=60) being male and primarily originating from urban areas (n=94; 86.2%). Gastrointestinal bleeding (n=74; 67.9%) was the main clinical manifestation, followed by intestinal obstruction and acute abdominal pain (n=10; 9.2% for both cases). At the time of diagnosis, the majority of subjects had a normal weight (n=84; 77.1%). The most common personal history was smoking (n=30; 27.5%), followed by alcoholism (n=20; 18.3%), and only 10 individuals had a family history of CRC (**Table 1**).

**Table 1:** Baseline characteristics of the study population (N=109).

	n	%
**Age** (years), mean (SD)	69.95 (13.49)	–
Gender		
Male	60	55
Female	49	45
Race		
Indigenous	5	4.6
African descent	29	26.6
White	25	22.9
Mestizo	15	13.8
Mulatto	35	32.1
Origin		
Urban	94	86.2
Rural	15	13.8
Main clinical manifestation		
Intestinal obstruction	10	9.2
Acute abdominal pain	10	9.2
Change in bowel habits	5	4.6
Gastrointestinal bleeding	74	67.9
Unintentional weight loss	5	4.6
Asymptomatic	5	4.6
Body Mass Index		
Underweight	20	18.3
Normal weight	84	77.1
Overweight/Obesity	5	4.6
Personal history		
Smoking	30	27.5
Alcoholism	20	18.3
Type 2 Diabetes Mellitus	10	9.2
Polyps	10	9.2
Family history		
Colorectal cancer	10	9.2

SD, Standard deviation.

Adenocarcinoma (n=104; 95.4%) was the predominant histological tumor type, with the middle third of the rectum being the most frequent location (n=69; 63.3%). Approximately 63.3% of the tumors were undifferentiated, while metastasis was identified in a mere 9.2% of the cases (n=10). The most frequent staging classifications were clinical-pathological stages IIIB and I (n=39; 35.8% and n=25; 22.9%, respectively), pathological stage III (n=64; 58.7%), regional/advanced clinical stage (n=64; 58.7%), and advanced progression stage (n=74; 67.9%) (**Table 2**).

**Table 2:** Oncological characteristics and molecular expression of the study population (N=109).

	n	%
Histopathological diagnosis		
Adenocarcinoma	104	95.4
Squamous cell carcinoma	5	4.6
Tumor location		
Middle third of the rectum	69	63.3
Distal third of the rectum	40	36.7
Degree of differentiation		
Undifferentiated	69	63.3
Well-differentiated	40	36.7
Metastasis		
Yes	10	9.2
No	99	90.8
Clinical-pathological staging		
Stage 1	25	22.9
Stage IIC	10	9.2
Stage IIIA	15	13.8
Stage IIIB	39	35.8
Stage IIIC	10	9.2
Stage IVA	5	4.6
Stage IVB	5	4.6
Pathological staging		
Stage I	25	22.9
Stage II	10	9.2
Stage III	64	58.7
Stage IV	10	9.2
Clinical stage		
Local/Early	35	32.1
Regional/Advanced	64	58.7
Metastatic/Advanced	10	9.2
Progression stage		
Early	35	32.1
Advanced	74	67.9
**Underwent radiotherapy**	10	9.2
**Pre-quantitative OCT4,** median (IQR)	17,998,731 (19,967,288 – 12,471,602)	–
**Pre-quantitative GUSB,** median (IQR)	23,230,681 (25,453,832 – 21,802,539)	–
**Pre-quantitative OCT4/GUSB,** median (IQR)	0.7657 (0.9026 – 0.4676)	–
**Post-quantitative OCT4,** median (IQR)	18,432,015 (21,641,436 – 13,003,910)	–
**Post-quantitative GUSB,** median (IQR)	23,094,681 (26,487,931 – 21,967,978)	–
**Post-quantitative OCT4/GUSB,** median (IQR)	0.763 (0.9034 – 0.5017)	–
**Pre-CEA, (ng/ml),** median (IQR)	3.12 (1.92 – 5.93)	–
**Post-CEA, (ng/ml),** median (IQR)	4.16 (1.75 – 7.65)	–
**ΔCEA**, (ng/ml), median (IQR)	-1.03 (-3.82 – 0.84)	–
**ΔOCT4**, median (IQR)	-873,452 (319,346 - -1,758,828	–
**ΔOCT4/GUSB**, median (IQR)	-0,0186 (0,0247 - -0,0474)	–
OCT4 expression		
Positive	84	77.1
Negative	25	22.9
ΔCEA		
Elevated (< -0.94)	56	51.4
Normal (> -0.94)	53	48.6
ΔOCT4 value		
Negative (no response)	78	71.6
Positive (response observed)	31	28.4
ΔOCT4/GUSB value		
Negative (no response)	74	67.9
Positive (response observed)	35	32.1
Elevated ΔCEA		
OCT4 positive expression	54	49.5
ΔOCT4 negative value (no response)	55	50.5
ΔOCT4/GUSB negative value (no response)	54	49.5

*CEA, carcinoembryonic antigen; IQR, interquartile range.

**Figure 1:**
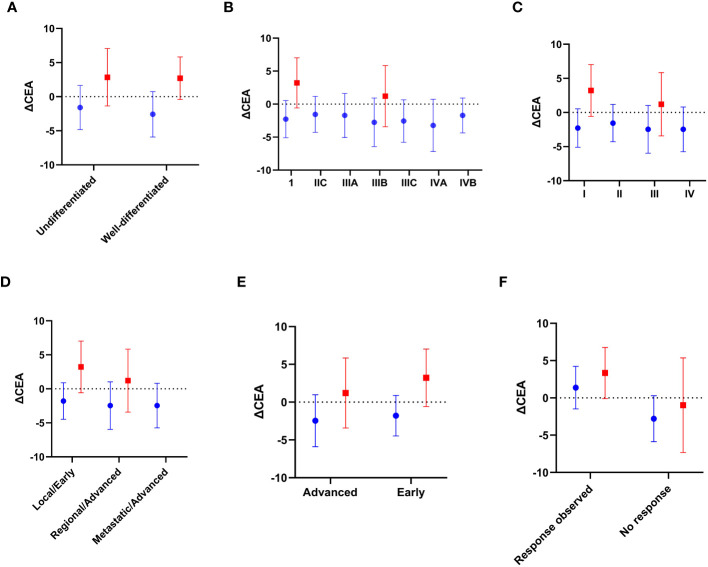
Blue: OCT4 positive expression; Red: OCT4 negative expression. Mean value differences of ΔCEA based on: **(A)** Degree of differentiation; **(B)** Clinical-pathological stage; **(C)** Pathological stage; **(D)** Clinical stage; **(E)** Progression stage; and **(F)** Therapeutic response (ΔOCT4 positive value vs. ΔOCT4 negative value, respectively), stratified according to the initial expression of the OCT4-cancer stem cell marker. Source: authors.

Concerning the expression status of the OCT4 cancer stem cell marker, initial positive expression was observed in 77.1% of the patients (n=84), with a median global expression prior to chemotherapy of 17,998,731 (IQR 12,471,602 - 19,967,288) among all samples. After chemotherapy, the median expression was 18,432,015 (IQR 13,003,910 - 21,641,436). Regarding CEA, the median levels before and after chemotherapy were 3.12 ng/ml (IQR 1.92 - 5.93) and 4.16 ng/ml (IQR 1.75 - 7.65), respectively. Thus, the median ΔCEA was -1.03 (-3.82 - 0.84) ng/ml, with elevated levels found in 51.4% of the subjects (n=56) (**Figure 1**, **Table 2**).

### 3.2 Association of ΔCEA with clinical behavior and tumor phenotype

The higher value of ΔCEA was observed in male patients (-1.37; IQR 4.15), mulatto race (-2.23; IQR 4.39), asymptomatic (-1.79; IQR 8), with type 2 diabetes mellitus (-2.12; IQR 4.97), and overweight (-2.02; IQR 8.33). However, statistically significant differences in the median of ΔCEA were found in the following scenarios: undifferentiated tumors (-2.03; p=0.002), clinical-pathological stage IIIA tumors (p=0.003), IIIB tumors (p <0.001), and IIIC tumors (p=0.003) compared to stage I; pathological stage III tumors (p <0.001) and IV tumors (p <0.001) compared to stage I; regional/advanced clinical stage tumors (p <0.001) and metastatic/advanced stage tumors (p=0.024) compared to stage I; and in the progression stage (advanced cancer vs. early; p <0.001) (**Table 3**).

**Table 3:** Associations between sociodemographic, clinical, and oncological variables with the ΔCEA value (N=109).

	Median	IQR	p-value
**Gender, mean (SD)**			0.59
Male	-1.37	4.15	
Female	-0.89	4.01	
**Race**			0.81
Indigenous	0.11	7.40	
African descent	-0.29	4.43	
White	-0.89	5.33	
Mestizo	-1.78	4.45	
Mulatto	-2.23	4.39	
**Origin**			0.22
Urban	-1.14	4.51	
Rural	-0.2	7.04	
**Main clinical manifestation**			0.93
Intestinal obstruction	-0.93	4.42	
Acute abdominal pain	-1.32	6.45	
Change in bowel habits	0.40	9.46	
Gastrointestinal bleeding	-1.02	4.79	
Unintentional weight loss	-1.20	6.12	
Asymptomatic	-1.79	8	
**Body Mass Index**			0.70
Underweight	-1.13	4.44	
Normal weight	-0.96	4.87	
Overweight/Obesity	-2.02	8.33	
Personal history			
Smoking	-1.11	4.69	0.85
Alcoholism	-1.58	4.45	0.52
Type 2 Diabetes Mellitus	-2.12	4.97	0.38
Polyps	-1.86	5.80	0.31
Family history			
Colorectal cancer	-0.08	6.56	0.33
**Histopathological diagnosis**			0.68
Adenocarcinoma	-1.02	4.76	
Squamous cell carcinoma	-1.23	6.18	
**Tumor location**			0.06
Middle third of the rectum	0.70	4.48	
Distal third of the rectum	-1.79	4.46	
**Degree of differentiation**			0.002*
Undifferentiated	-2.03	4.33	
Well-differentiated	0.42	6.36	
**Metastasis**			0.33
Yes	-1.01	4.74	
No	-2.06	4.57	
**Clinical-pathological staging**			0.002*
Stage 1	1.14	4.50	
Stage IIC	-1.86	4.43	
Stage IIIA^a^	-2.1	4.27	
Stage IIIB^b^	-2.03	4.65	
Stage IIIC^c^	-2.14	4.56	
Stage IVA	-2.34	6.66	
Stage IVB	-1.79	5.09	
**Pathological staging**			<0.001*
Stage I	1.14	4.50	
Stage II	-1.86	4.43	
Stage III^d^	-2.07	4.44	
Stage IV^e^	-2.06	4.57	
**Clinical stage**			<0.001*
Local/Early	0.61	5.53	
Regional/Advanced^f^	-2.07	4.44	
Metastatic/Advanced^g^	-2.06	4.57	
**Progression stage**			<0.001*
Early	0.61	5.53	
Advanced	-2.07	4.38	
**Underwent radiotherapy**	-1.74	5.82	0.34
**OCT4 expression**			<0.001*
Positive	-2.16	4.17	
Negative	1.26	4.31	
**ΔOCT4 value**			<0.001*
Positive	1.26	3.97	
Negative	-2.29	3.73	
**ΔOCT4/GUSB value**			<0.001*
Positive	1.19	4.10	
Negative	-2.4	3.36	

*Statistical significance: <0.05.

a Statistically significant difference compared to stage I (p=0.003).

b Statistically significant difference compared to stage I (p <0.001).

c Statistically significant difference compared to stage I (p=0.003).

d Statistically significant difference compared to stage I (p=0.004).

e Statistically significant difference compared to stage I (p <0.001).

f Statistically significant difference compared to local/early stage (p <0.001).

g Statistically significant difference compared to local/early stage (p=0.024).

**Figure 2:**
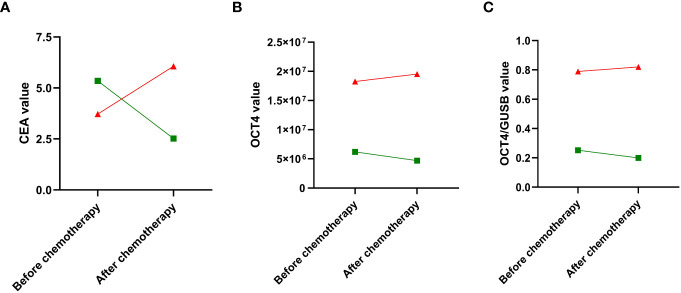
Red: OCT4 positive expression; Green: OCT4 negative expression. Change in the absolute values of the quantitative parameters: **(A)** CEA; **(B)** OCT4; and **(C)** OCT4/GUSB, before and after chemotherapy, stratified according to the initial expression of the OCT4-cancer stem cell marker. Source: authors.

Furthermore, significant differences in the median values of ΔCEA were observed based on the initial expression status of the OCT4 marker in tumors (p <0.001), as well as in the values of ΔOCT4 (p <0.001) and ΔOCT4/GUSB (p <0.001) (**Figure 2**). When examining the categorical values of ΔCEA (elevated vs. normal), a statistically significant association was found between elevated ΔCEA and tumor undifferentiation (p=0.008), clinical-pathological stage IIIB (p=0.006), pathological stage III (p <0.001), locally/advanced clinical stage (p=0.004), positive expression of OCT4 at the time of diagnosis (p <0.001), and negative values of ΔOCT4 (p <0.001) and ΔOCT4/GUSB (p <0.001), indicating a poor therapeutic response (**Table 4**).

**Table 4:** Associations between gender, oncological variables, and molecular expression with ΔCEA category (N=109).

Variable	Elevated ΔCEA (n=56)	Normal ΔCEA (n=53)	p-value
	n (%)		
**Gender**			0.44
Male	33 (58.9)	27 (50.9)	
Female	23 (41.1)	26 (49.1)	
**Tumor location**			0.53
Middle third of the rectum	40 (71.4)	29 (54.7)	
Distal third of the rectum	16 (28.6)	24 (55.3)	
**Degree of differentiation**			0.008*
Undifferentiated	42 (75)	27 (50.9)	
Well-differentiated	14 (25)	26 (49.1)	
**Metastasis**			0.40
Yes	6 (89.3)	4 (7.5)	
No	50 (10.7)	49 (92.5)	
Clinical-pathological staging			
Stage 1	4 (7.1)	21 (39.6)	
Stage IIC	6 (10.7)	4 (7.5)	
Stage IIIA	9 (16.1)	6 (11.3)	0.006*
Stage IIIB	25 (44.6)	14 (26.4)	
Stage IIIC	6 (10.7)	4 (7.5)	
Stage IVA	3 (5.4)	2 (3.8)	
Stage IVB	3 (5.4)	2 (3.8)	
Pathological staging			
Stage I	4 (7.1)	21 (39.6)	
Stage II	6 (10.7)	4 (7.5)	<0.001*
Stage III	40 (71.4)	24 (45.3)	
Stage IV	6 (10.7)	4 (7.5)	
Clinical stage			
Local/Early	10 (17.9)	25 (47.2)	0.004*
Regional/Advanced	40 (71.4)	24 (45.3)	
Metastatic/Advanced	6 (10.7)	4 (7.5)	
**Progression stage**			<0.001*
Early	10 (17.9)	25 (47.2)	
Advanced	46 (82.1)	28 (52.8)	
**OCT4 expression**			<0.001*
Positive	54 (96.4)	30 (56.6)	
Negative	2 (3.6)	23 (43.4)	
**ΔOCT4 value**			<0.001*
Positive	1 (1.8)	10 (43.4)	
Negative	55 (98.2)	23 (56.6)	
**ΔOCT4/GUSB value**			<0.001*
Positive	2 (3.6)	33 (62.3)	
Negative	54 (96.4)	20 (37.7)	

*Statistical significance: <0.05.

CEA: carcinoembryonic antigen.

### 3.3 Elevated ΔCEA in rectal cancer with OCT4-positive cancer stem cells

Being OCT4 positive and having an elevated ΔCEA value were significantly associated with undifferentiated tumor phenotype (PR 2.02; 95% CI: 1.21 - 3.37, p=0.002), advanced tumor progression stage (PR 2.36; 95% CI: 1.30 - 4.27, p <0.001), and negative values of ΔOCT4 (PR 21.06; 95% CI: 3.04 - 145.7, p <0.001) and ΔOCT4/GUSB (PR 12.29; 95% CI: 3.17 - 47.61, p <0.001) (suggestive of poor therapeutic response) compared to those without this status. **Table 5** presents additional associations that can be reviewed.

**Table 5:** Association between elevated ΔCEA + OCT4 positive expression status with oncological variables of interest (N=109).

	Elevated ΔCEA + OCT4 positive expression n (%)		p-value
	Yes (n=54)	No (n=55)	
**Gender**			0.14
Male	33 (61.1)	27 (49.1)	
Female	21 (38.9)	28 (50.9)	
**Tumor location**			0.04*
Middle third of the rectum	39 (72.2)	30 (54.5)	
Distal third of the rectum	15 (27.8)	25 (45.5)	
**Degree of differentiation**			0.002*
Undifferentiated	42 (77.8)	27 (49.1)	
Well-differentiated	12 (22.2)	28 (50.9)	
**Metastasis**			0.35
Yes	6 (11.1)	4 (7.3)	
No	48 (88.9)	51 (92.7)	
Clinical-pathological staging			
Stage 1	3 (5.6)	22 (40)	
Stage IIC	6 (11.1)	4 (7.3)	
Stage IIIA	9 (16.7)	6 (10.9)	
Stage IIIB	24 (44.4)	15 (27.3)	0.002*
Stage IIIC	6 (11.1)	4 (7.3)	
Stage IVA	3 (5.6)	2 (3.6)	
Stage IVB	3 (5.6)	2 (3.6)	
Pathological staging			
Stage I	3 (5.6)	22 (40)	
Stage II	6 (11.1)	4 (7.3)	<0.001*
Stage III	39 (72.2)	25 (45.5)	
Stage IV	6 (11.1)	4 (7.3)	
**Clinical stage**			0.003*
Local/Early	9 (16.7)	26 (47.3)	
Regional/Advanced	39 (72.2)	25 (45.5)	
Metastatic/Advanced	6 (11.1)	4 (7.3)	
**Progression stage**			<0.001*
Early	9 (16.7)	26 (52.7)	
Advanced	45 (83.3)	29 (47.3)	
**ΔOCT4 value**			<0.001*
Negative	53 (98.1)	25 (45.4)	
Positive	1 (1.9)	30 (54.5)	
**ΔOCT4/GUSB value**			<0.001*
Negative	52 (96.3)	22 (40)	
Positive	2 (3.7)	33 (60)	

*Statistical significance: <0.05.

CEA, carcinoembryonic antigen.

Significant associations were found in cases with elevated ΔCEA values and negative ΔOCT4 (indicative of progression and poor therapeutic response), including undifferentiated tumor phenotype (PR 1.69; 95% CI: 1.06 - 2.70, p=0.012), advanced tumor progression stage (PR 2.12; 95% CI: 1.22 - 3.70, p=0.002), and initial positive expression of OCT4 (PR 7.88; 95% CI: 2.08 - 30.1, p <0.001). Other associations are visualized in **Table 6**.

**Table 6:** Association between elevated ΔCEA + ΔOCT4 negative value status with oncological variables of interest (N=109).

Variable	Elevated ΔCEA + ΔOCT4 negative value n (%)		p-value
	Yes (n=55)	No (n=54)	
**Gender**			0.31
Male	32 (58.2)	28 (51.9)	
Female	23 (41.8)	26 (48.1)	
**Tumor location**			0.07
Middle third of the rectum	39 (70.9)	30 (55.6)	
Distal third of the rectum	16 (29.1)	24 (44.4)	
**Degree of differentiation**			0.012*
Undifferentiated	41 (74.5)	28 (51.9)	
Well-differentiated	14 (25.5)	28 (48.1)	
**Metastasis**			0.38
Yes	6 (10.9)	4 (7.4)	
No	49 (89.1)	50 (92.6)	
Clinical-pathological staging			
Stage 1	4 (7.4)	21 (38.9)	
Stage IIC	6 (10.9)	4 (7.4)	
Stage IIIA	9 (16.4)	6 (10.9)	
Stage IIIB	24 (43.6)	15 (27.8)	0,009*
Stage IIIC	6 (10.9)	4 (7.4)	
Stage IVA	3 (5.5)	2 (3.7)	
Stage IVB	3 (5.5)	2 (3.7)	
Pathological staging			
Stage I	4 (7.4)	21 (38.9)	
Stage II	6 (10.9)	4 (7.4)	<0.001*
Stage III	39 (70.9)	25 (46.3)	
Stage IV	6 (10.9)	4 (7.4)	
**Clinical stage**			0.006*
Local/Early	10 (18.2)	25 (46.3)	
Regional/Advanced	39 (70.9)	25 (46.3)	
Metastatic/Advanced	6 (10.9)	4 (7.4)	
**Progression stage**			0.002*
Early	10 (18.2)	29 (53.7)	
Advanced	45 (81.8)	25 (46.3)	
**OCT4 expression**			<0.001*
Positive	53 (96.4)	31 (57.4)	
Negative	2 (3.6)	23 (42.6)	

*Statistical significance: <0.05.

CEA, carcinoembryonic antigen.

Similarly, having an elevated ΔCEA and negative ΔOCT4/GUSB were significantly associated with undifferentiated tumor phenotype (PR 1.82; 95% CI: 1.12 - 2.97, p=0.006), advanced tumor progression stage (PR 2.36; 95% CI: 1.30 - 4.27, p <0.001), and initial positive expression of OCT4 (PR 7.73; 95% CI: 2.02 - 29.54, p <0.001) compared to not having this status (**Table 7**).

**Table 7:** Association between elevated ΔCEA + ΔOCT4/GUSB negative value status with oncological variables of interest (N=109).

	Elevated ΔCEA + ΔOCT4/GUSB negative value n (%)		p-value
	Yes (n=54)	No (n=55)	
**Gender**			0.24
Male	32 (59.3)	28 (50.9)	
Female	22 (40.7)	27 (49.1)	
**Tumor location**			0.017*
Middle third of the rectum	40 (74.1)	29 (52.7)	
Distal third of the rectum	14 (25.9)	26 (47.3)	
**Degree of differentiation**			0.006*
Undifferentiated	41 (75.9)	28 (50.9)	
Well-differentiated	13 (24.1)	27 (49.1)	
**Metastasis**			0.35
Yes	6 (11.1)	4 (7.3)	
No	48 (88.9)	51 (92.7)	
Clinical-pathological staging			
Stage 1	3 (5.6)	22 (40)	
Stage IIC	6 (11.1)	4 (7.3)	
Stage IIIA	9 (16.7)	6 (11.1)	
Stage IIIB	24 (44.4)	15 (27.3)	0,002*
Stage IIIC	6 (11.1)	4 (7.3)	
Stage IVA	3 (5.6)	2 (3.6)	
Stage IVB	3 (5.6)	2 (3.6)	
Pathological staging			
Stage I	3 (5.6)	22 (40)	
Stage II	6 (11.1)	4 (7.3)	<0.001*
Stage III	39 (72.2)	25 (45.5)	
Stage IV	6 (11.1)	4 (7.3)	
**Clinical stage**			0.003*
Local/Early	9 (16.7)	26 (47.3)	
Regional/Advanced	39 (72.2)	25 (45.5)	
Metastatic/Advanced	6 (11.1)	4 (7.3)	
**Progression stage**			<0.001*
Early	9 (16.7)	26 (47.3)	
Advanced	45 (83.3)	29 (52.7)	
**OCT4 expression**			<0.001*
Positive	52 (96.3)	32 (58.2)	
Negative	2 (3.7)	33 (41.8)	

*Statistical significance: <0.05.

CEA, carcinoembryonic antigen.

The presence of an elevated ΔCEA in conjunction with OCT4 positivity was found to exhibit a significant association with negative values of ΔOCT4 and ΔOCT4/GUSB (p <0.001 for both cases) when compared to OCT4 negativity, suggesting a potential link to a suboptimal therapeutic response (**Table 8**). Lastly, upon evaluating the linear regression analysis examining the relationship between quantitative OCT4 expression values and the deltas of OCT4 and OCT4/GUSB with ΔCEA, it was determined that while all three variables showed a statistically significant association with predicting the outcome (p <0.001 in all cases), the highest R^2^ value attained was only 0.42 (in the case of ΔOCT4). Furthermore, this association was also observed in the correlation analysis of the quantitative values between ΔCEA and OCT4 (Rho -0.45; p <0.001), ΔOCT4 (Rho 0.65; p <0.001), and ΔOCT4/GUSB (Rho 0.61; p <0.001).

**Table 8:** Association between categories of ΔCEA and ΔOCT4 and ΔOCT4/GUSB, according to OCT4 expression status (N=109).

	OCT4 positive expression (N=84)		p-value	OCT4 negative expression (N=25)		p-value
	Elevated ΔCEA	Normal ΔCEA		Elevated ΔCEA	Normal ΔCEA	
	n (%)			n (%)		
ΔOCT4						
Negative	53 (98.1)	22 (73.3)	<0.001*	2 (100)	1 (4.3)	0.10
Positive	1 (1.9)	8 (26.7)	0	22 (95.7)
ΔOCT4/GUSB						
Negative	52 (96.3)	18 (60)	<0.001*	2 (100)	2 (8.7)	0.02*
Positive	2 (3.7)	12 (40)	0	21 (91.3)

*Statistical significance: <0.05.

CEA, carcinoembryonic antigen.

## 4 Discussion

The comprehension of the tumor microenvironment, gITH (13), and genotype-phenotype correlation (11) in cancer research establishes a fundamental basis for the identification of targets that permit precise, safe, and effective inhibition of tumorigenesis, migration, differentiation, metastasis, and therapeutic resistance in cancer (10, 11, 13, 14). CSCs represent a crucial element in the tumor microenvironment, demonstrating an unimaginable adaptive capacity against external agents that favors the development of aggressive phenotypes, rapid progression, and a poor therapeutic response (34, 35). While numerous studies have identified potential molecular biomarkers in diverse cancers, the genetic diversity of populations and the phenotype resulting from the interaction between the environment and genotype/epigenotype hinder the potential extrapolation of these molecular biomarkers (36). In particular, limited evidence exists in RC regarding molecular biomarkers indicating CSC expression, which could be utilized for personalized chemotherapy treatment in individuals with a more aggressive tumor biology and phenotype. The universal expression of OCT4 in CSCs (as a biological regulator of these cells) has been acknowledged, enabling the differentiation of tumors with pluripotency from those without, ultimately leading to distinct clinical behavior and a worse prognosis (16).

Considering that OCT4 expression could determine the tumor phenotype and therapeutic response in RC (37), how can the evolution and chemotherapy response of RC with CSCs positive expression be evaluated in clinical practice? This assessment can be achieved using a clinically utilized biomarker with quantitative expression dependent on the tumor phenotype and progression. The hypothesis that the quantification of CEA, a glycoprotein whose expression possibly is regulated by oncofetal genes like OCT4, is directly and significantly associated with the quantitative expression of OCT4 in RC has been confirmed by this study. Moreover, the persistent association observed after chemotherapy (represented by the values of ΔOCT4 and ΔCEA, indicating the change before and after chemotherapy) provides evidence of its correlation as a clinical biomarker for monitoring and assessing therapeutic response. This finding could help enhance the prognostic performance of RC through the use of combined biomarkers. This is due to the fact that CEA by itself exhibits insufficient performance in predicting the prognosis of RC (sensitivity of 50% and specificity below 80%) (38).

Initially, it was observed that RC exhibited sociodemographic and clinical behavior similar to the literature reports (17–19, 39). It tends to occur more frequently in older adults (around 60-70 years old) probably due to prolonged exposure to risk factors throughout their lives (40). It also affects individuals from ethnic groups considered vulnerable and at high risk in certain regions (41). For instance, individuals of African descent and Mulattos represented 29 (26.6%) and 35 (32.1%) cases respectively, which can be attributed to socioeconomic inequities, limited healthcare access, and unhealthy lifestyles resulting from difficulties in accessing health education (41). The primary manifestation of RC was gastrointestinal bleeding (n=74; 67.9%), which could be explained by localized anatomical abnormalities in the rectum. Among the sampled individuals, 77.1% (n=84) had a normal weight range. Although the reported prevalence of overweight and obesity in the Caribbean region of Colombia is significant (42), this can be explained by unreported weight loss at the time of diagnosis. Furthermore, approximately 30% and 20% reported being smokers and frequent alcohol consumers, respectively. These behaviors constitute potential risk factors for cancer, including RC (43). Interestingly, only 9.2% reported a family history of colorectal cancer, suggesting a lack of significant association between cancers related to familial patterns in this region.

While adenocarcinoma (95.4%) was the most frequent histological diagnosis, the undifferentiated phenotype (n=69; 63.3%) predominated, with metastasis occurring in only 10 cases at the time of diagnosis. This suggests a strong influence of genotype-phenotype correlation on the tumorigenesis of the rectal tissue cell line of tumor origin (11). The heterogeneous, distinct, and more aggressive pattern of differentiation observed (possibly due to alterations in regulatory markers like OCT4) may explain this phenomenon (16). However, migration is limited, likely due to the absence or inactivation of a necessary cofactor for this process (16, 34). On the other hand, invasion behavior is more frequent, which accounts for the predominance of stage III (n=64; 58.7%) and advanced stage (n=74; 67.9%) at the time of diagnosis. Regarding molecular expression during OCT4 diagnosis, positivity was observed in 77.1% (n=84), which explains the predominant behavior of other described clinical and staging variables, such as the undifferentiated phenotype and advanced stage. Importantly, this prevalence correlates with the percentage of individuals who showed negative values for ΔOCT4 (71.6%) and ΔOCT4/GUSB (67.9%), indicating a poor therapeutic response. This implies a direct relationship between the initial identification of CSC-positive RC and the expected therapeutic response.

When we compare our results with those reported in the literature so far, we find that, although fewer than five studies have specifically investigated the relationship between OCT4 and RC (and none to date between OCT4 and CEA) (17–19, 39), similar evidence suggests a comparable clinical and sociodemographic behavior. These studies have revealed a male/female prevalence ratio > 1, with cancer often presenting between the ages of 60-70 years, a predominance of moderate to poor degree of differentiation, and a high frequency of stage III cases at the time of diagnosis. It is noteworthy that all of these studies originate from the Asian continent (China, Japan, and Iran) (17, 19, 39), and the similarities could be explained by the influence of certain risk factors throughout life in colon cancer and RC. The expression of OCT4 in an alternative form of gastrointestinal cancer has been investigated by several other studies, revealing a comparable trend in relation to the prognostic potential of OCT4 (44). Nevertheless, a correlation with CEA has not been established in these findings, and the study cohort has comprised patients subjected to surgical resection. In our instance, the study population included individuals who had not undergone surgical resection (factor directly impacting the cancer’s behavior).

Finally, we found a plausible and expected relationship between the medians of ΔCEA and the degree of differentiation, staging, initial expression of OCT4, ΔOCT4, and ΔOCT4/GUSB, indicating significantly lower ΔCEA values (i.e., post-chemotherapy CEA value higher than the baseline value). This relationship can be explained by tumor plasticity and cellular phenotype, which may exhibit hyperplasia and lack of differentiation in the original cell line. This leads to increased expression of the CEA glycoprotein in tumors with larger size, greater tissue invasion, and poorer degrees of differentiation. These findings support the self-renewal and pluripotency properties observed in tumors with CSCs positive expression, ultimately resulting in rapid progression that hampers early-stage cancer diagnosis. When grouping the elevated ΔCEA category status (< -0.94 ng/ml) with OCT4 positive expression, these associations persist compared to not having this status. In other words, we can observe a direct proportionality between CEA expression and OCT4 expression. In cases with a poor therapeutic response, where OCT4 expression continues to increase due to cancer progression, an increase in CEA values is also expected. This leads to negative ΔCEA values. Therefore, ΔCEA could serve as a clinical biomarker for monitoring tumor behavior and therapeutic response in RC. Then, it correlates directly with the expression of OCT4, ΔOCT4, and ΔOCT4/GUSB.

These results offer various translational applications: 1) Evaluating the initial expression of the OCT4 marker in RC samples would be interesting and valuable to determine CSCs positivity in the tumor and consider the potential persistence of pluripotency and tumor plasticity properties. 2) Developing an algorithm based on CSC expression status could establish a personalized approach to assess the impact of curative or palliative surgery, radiotherapy, and chemotherapy according to the specific therapeutic regimens employed. 3) Seeking to predict the prognosis and survival of individuals with RC and CSCs positive expression could inform a multidisciplinary approach and shorter intervals between chemotherapy sessions or additional drugs targeting specific signaling pathways related to CSCs. 4) Considering the risk of adverse events and quality of life in individuals with a poor expected therapeutic response becomes crucial, as chemotherapy significantly impacts the physical and emotional well-being of the patient. Furthermore, identifying specific pathways in these tumors with OCT4 expression could facilitate the use of targeted biological agents to inhibit frequently expressed signaling pathways involved in differentiation, invasion, and self-renewal. This has the potential to significantly impact the survival of affected individuals. Additionally, the fundamental novelty and pertinence of our research hypothesis are rooted in the absence of any description, up until the present moment, of an alternative serum biomarker that may be amalgamated with CEA to amplify diagnostic performance. Nevertheless, the potential for conjunction arises with a molecular examination, exemplified by the OCT4 status.

We acknowledge the small sample size as a limitation, which may introduce biases when comparing different subgroups based on OCT4 expression and ΔCEA status. Additionally, the exploratory design did not allow for evaluating changes in clinical and oncological parameters after chemotherapy and their correlation with ΔCEA, ΔOCT4, and ΔOCT4/GUSB values. Furthermore, other post-chemotherapy follow-up parameters that could provide a more precise assessment of cancer progression were not included. However, we want to highlight the following strengths of this study: 1) This study is the first to evaluate OCT4 expression in fresh RC samples, unlike previous studies (17, 19, 39), which enhances the quality of molecular analysis and result accuracy; 2) It established a correlation between the expression of OCT4 and CEA markers in RC; 3) It analyzed changes in OCT4 and CEA expression before and after chemotherapy, providing novel evidence of a potential routine biomarker for RC monitoring in clinical practice; 4) This study established a new association between two potential signaling pathways linked to the germ cell line of rectal tissue (related to CEA and OCT4 expression in the mutagenic and neoplastic state). These findings lay the groundwork for future studies aiming to conduct cell cultures to describe the signaling pathways associated with differentiation, migration, invasion, self-renewal, and other cellular properties in tumor cells with CSCs positive expression. This can lead to the development of new hypotheses or a more precise understanding of the behavior and tumor microenvironment in RC among individuals with these clinical, oncological, and sociodemographic characteristics.

## 5 Conclusions

This study identified a significant and directly proportional association among the values of ΔCEA, ΔOCT4, and ΔOCT4/GUSB. These findings suggest that ΔCEA holds potential as a clinical biomarker for determining the undifferentiated tumor phenotype, advanced clinical stage, and poor therapeutic response in RC patients with positive expression of the OCT4 CSCs marker.

## Data availability statement

The original contributions presented in the study are included in the article/supplementary materials, further inquiries can be directed to the corresponding authors.

## Ethics statement

The studies involving humans were approved by Ethics Committee of Facultad de Medicina - Universidad de Cartagena. The studies were conducted in accordance with the local legislation and institutional requirements. The participants provided their written informed consent to participate in this study.

## Author contributions

IL-M: Conceptualization, Data curation, Formal Analysis, Investigation, Methodology, Writing – original draft, Writing – review & editing. MB-R: Conceptualization, Data curation, Formal Analysis, Investigation, Methodology, Writing – original draft, Writing – review & editing. LL-A: Conceptualization, Data curation, Formal Analysis, Investigation, Methodology, Writing – original draft, Writing – review & editing. YL: Conceptualization, Data curation, Investigation, Methodology, Writing – original draft, Writing – review & editing. AS-C: Conceptualization, Data curation, Investigation, Methodology, Writing – original draft, Writing – review & editing.
